# Influences of perfectionism and motivational climate on attitudes towards doping among Korean national athletes: a cross sectional study

**DOI:** 10.1186/s13011-017-0138-x

**Published:** 2017-12-12

**Authors:** Moonjung Bae, Jungjoong Yoon, Hyunyong Kang, Taegyu Kim

**Affiliations:** 1 Department of Sports Medicine and Science, Taereung National Training Center of the Korean Sport & Olympic Committee, 727, Hwarang-ro, Nowon-gu, Seoul, 01794 Republic of Korea; 2 Department of Marine Sports, Pukyong National University, 45, Yongso-ro, Nam-Gu, Busan, 48513 Republic of Korea

**Keywords:** Performance enhancement, Doping behavior, Elite athlete, Rio Olympics

## Abstract

**Background:**

The motives for elite athletes to dope are related primarily to maintaining and improving their physical performance. Especially, elite athletes training to compete in the Olympics may feel unique situational pressure, which may in turn induce powerful motivation for doping and predict doping behavior. This study aimed to investigate possible factors associated with attitudes towards doping in Korean national athletes who competed in the Rio 2016 Olympic Games.

**Methods:**

A total of 198 athletes (95 female, 103 male) completed the questionnaire, which covered demographic information, doping-related experiences, Performance Enhancement Attitude Scale (PEAS), Perfectionism in Sports Scale (PSS; coach’s criticism, concern over mistakes, and personal standards), and Perceived Motivational Climate in Sport Questionnaire-2 (PMCSQ-2; ego-involving and task-involving climates). Pearson’s correlation coefficients were used to identify correlations among PEAS, PSS, and PMCSQ-2 scores, and stepwise multiple linear regression was performed to investigate possible factors significantly associated with attitudes towards doping.

**Results:**

The coach’s criticism of PSS was slightly or weakly related to the concern over mistakes of PSS and the ego-involving climate of PMCSQ-2, respectively. And the concern over mistakes sub-scale of perfectionism was related to attitudes towards doping, but weakly.

**Conclusions:**

Effective anti-doping policy should meet athletes’ perfectionism, and more studies that identify other factors that influence athletes’ doping attitudes are needed.

## Background

Doping formally refers to any violation of anti-doping rules set forth in the Code of the World Anti-Doping Agency (WADA) [1]. It hurts the image of sports, is considered unsportsmanlike, and poses potentially irreversible health consequences to users [2, 3]. The efforts of the WADA and national anti-doping agencies to prevent doping, including serious disciplinary consequences for athletes who are caught, have led to rising awareness of doping and more frequent application of controls [4]. However, the rate of positive doping tests remains around 2% [5] and self-administrated surveys show that real rates of doping may lie between 3% and 15% [6], meaning that the doping phenomenon is still pervasive [7]. In 2015, “Anti-Doping Testing Figures” published by WADA reported that of a total of 303,369 samples analyzed, 5912 positive results (1.95%) were found [1]. In the South Korean context specifically, in 2015, the Korean Anti-Doping Agency (KADA) analyzed 3782 samples and found 35 positive results, accounting for 0.9% of total samples [8].

The most common approach to prevention of doping has been a detection approach, involving regular doping controls and disciplinary consequences for positive tests, alongside a deterrence approach, consisting of anti-doping education [6]. However, detection is considered ineffective for prevention of doping because of the high cost of frequent doping controls and the emergence of new technologies for circumventing them [6, 9, 10]. In contrast to detection, a deterrence approach composed of anti-doping education, that is, a knowledge-based approach, is best complemented by a prevention approach [6, 9, 11]. It is because enough information on the risks and dangers of doping may change attitudes towards doping and/or lead to decrease in doping intention [6, 12, 13]. However, many studies suggest that simple knowledge-transfer via information about doping—what it is, that it is forbidden, and that it is unhealthy—is just not enough to change athletes’ attitudes and/or behaviors [6, 13–15]. Therefore, a deterrence approach should consider individual and situational psychological factors that influence athletes’ doping behaviors and attitudes towards doping [6, 16].

Doping is not an accident [7], and its causes are complex [3]. Petróczi and Aidman [17] proposed a life-cycle model of performance enhancement that enables a more effective intervention approach to doping. This model explains that doping behavior is an intentional, self-regulated, and goal-directed behavior associated with performance enhancement, but does not specify the underlying psychological processes [18]. It places the decision to dope in the context of six phases: choice, goal commitment, execution, feedback on goal attainment, goal evaluation/adjustment, and the decision to repeat the cycle or abandon it [6, 7, 19]. Along these phases, various risk factors may lead the athletes to engage in doping behaviors; the model divides these factors into three groups: personality-related factors (e.g., perfectionism), situational factors (e.g., access and availability of substances), and systemic factors (e.g., motivational climate, anti-doping policies) [6, 7]. According to Petróczi and Aidman [17], the combination of these risk factors constitutes doping attitude that means the athletes’ beliefs and dispositions towards doping behaviors.

Athletes’ doping attitudes are often used as a proxy for doping behaviors, because those who use banned drugs have more permissive attitudes towards doping than those who never dope [17]. Previous articles mention that although the effect size of the relationship between attitudes towards doping and doping behavior is small [20], attitudes influence doping practice indirectly via doping assumption [7, 21–23]. In other words, a rational doping mindset or a positive attitude towards doping behaviors may lead to and support the use of banned drugs [7, 24]. As an element predicting actual practice of doping [7, 24], attitudes toward doping should be investigated in order to identify the factors that affect them, which would constitute useful knowledge to inform efforts to reduce risk by developing policies at the individual and environmental levels [7, 16].

In sports, perfectionism is broadly defined by striving for flawlessness and setting excessively high performance standards [25]. It is a personality attribute that helps to achieve higher physical and motor performance by adapting to this trait [7, 26]; at the same time, however it is often regarded as a maladaptive affective responses, in that it may lead to stress, fatigue, eating disorders, and/or emotional problems [25, 27–29]. As Zucchetti et al. [7] have reported, Italian athletes with excessive perfectionism have more positive attitudes towards doping, and many researchers may suspect that perfectionism is among the personality-related factors that affect attitudes towards doping [30, 31]. Four particular aspects of perfectionism, namely perfectionistic striving, perfectionistic concerns, parental pressure to be perfect, and coach pressure to be perfect [32–35], may independently impact attitudes towards doping.

Motivational climate in sports consists of the external achievement expectations of coaches, parents, peers and/or fans, as perceived by athletes [17]. Motivational climate formed by coaches plays a role in athletes’ ego or task orientation in relation to subsequent choices and behaviors [17, 36]. While athletes with a task orientation consider their competence and success to be a matter of personal improvement and development, those with an ego orientation define it as outperforming others and winning [16]. In other words, a task orientation has consistently been associated with sportspersonship, and an ego orientation with lower levels of moral functioning, unsportspersonlike attitudes, and less mature moral reasoning [37]. Thus, as Petróczi and Aidman [17] mention, motivational climate is a critical systemic factor in doping: attitudes towards doping become more permissive with increase in ego orientation compared to task orientation [38].

Previous studies also show that knowledge about doping and its side effects is related to doping intentions and behavior, but the correlations are weak [20, 39]. Studies also show that older and male athletes are more susceptible to doping behavior than younger and female athletes [6], and suggest the possibility that personal contact with users of banned drugs is positively related to attitudes towards doping [7]. Moreover, different sports differ with regards to achievement goal and prevailing perception of motivational climate [16]; although Zucchetti et al. [7] showed that there was no difference in athletes’ attitudes towards doping between resistance sports (which require resistance as the main ability necessary to win, e.g., cycling, road racing, triathlon) and non-resistance sports (which demand substantial motor skills), Allen et al. [16] mentioned that compared with Scottish individual athletes, team athletes had stricter attitude towards doping and lower ego orientation. Further studies on doping should look at how individual and situational factors may act as protective or risk factors in doping [16].

The motives for elite athletes to dope are known to be related primarily to maintaining and improving their physical performance, coping with social and/or psychological pressures to perform, and striving for social and/or psychological goals [40]. It is also expected that elite athletes will face various situational pressures within their daily training routine [2]. Especially because winning a medal at the Olympic Games is often considered to be the most important event in an athletes’ life and the highest goal to which athletes can strive [3], elite athletes training to compete in the Olympics may feel unique situational pressure [40], which may in turn induce powerful motivation for doping and predict doping behavior [2, 41, 42]. Thus, this study aims to investigate possible factors associated with attitudes towards doping, leading to doping behaviors, among Korean national athletes who competed in the 2016 Olympic Games in Rio de Janeiro and to provide useful information that will facilitate anti-doping strategies for Korean national athletes.

## Methods

### Participants

A total of 204 Korean athletes (101 female, 103 male) who competed in 24 of 28 Olympic sports during the Rio 2016 Olympic Games were selected for this study. Six of them were adolescent (≤18 years old), and they were excluded because there might be subtle differences between adults (>18 years old) and adolescents in relation to factors that influence attitudes towards doping and doping behaviors [43]; thus, this study involved a total of 198 athletes (95 female, 103 males). Prior to recruitment, ethical approval was gained from the institution’s research ethics committee, and all athletes gave their informed consent to participate in this study. Table 1 shows the participants’ characteristics.

**Table 1:** Characteristics of participants

Variable	Category	N	Percent
Gender	Female	95	48.0
	Male	103	52.0
Type of event	Team	76	38.4
	Individual	122	61.6
Contact with doping users	Yes	10	5.1
	No	188	94.9
Knowledge on doping	Yes	141	71.2
	No	57	28.8
Education on doping	Yes	126	63.6
	No	72	36.4
	Mean	Standard deviation	
Age	26.02	4.17	
PEAS	13.66	5.62	
Coach’s criticism	22.25	4.93	
Concern over mistakes	23.38	4.76	
Personal standards	28.91	4.66	
Task-involving climate	64.89	9.92	
Ego-involving climate	44.44	10.27	

*PEAS* Performance Enhancement Attitude Scale

### Procedure

This study used a cross-sectional design to investigate possible factors associated with athletes’ attitudes towards doping, leading to doping behaviors. Several questionnaires were employed to serve the study’s aim: 1) a questionnaire containing items on athletes’ demographic information and doping-related experiences, 2) the Performance Enhancement Attitude Scale (PEAS), 3) the Perfectionism in Sports Scale (PSS), and 4) the Perceived Motivational Climate in Sport Questionnaire-2 (PMCSQ-2). The questionnaires were accompanied by brief descriptions of performance-enhancing and recreational substances/methods and a cover letter explaining the purpose of this study [44, 45]. All data were collected during 10 pre-competition days before the 2016 Olympic Games, via an interviewer-administered survey carried out by 25 well-trained medical staff attached to the Korean Olympic team, in order to help respondents better understand each question and to achieve higher response rates compared to a self-administered survey [46]. Social desirability bias produced by the interaction between interviewer and athlete was minimized through careful training and monitoring of staff by the authors [46, 47].

### Characteristics of athletes

Data were collected on athletes’ demographic information, the sport they represent, knowledge and education on doping, and contact with dopers, using the questionnaires proposed by Moran et al. [44] and translated by Kim and Kim [47]. Sports were divided into two sub-groups by discipline—individual or team [16], and for some events whose sub-group was ambiguous because they included various disciplines, a coach from each discipline was consulted and asked to select a sub-group [44, 47]. All participants were asked to respond yes or no to 3 questions on knowledge and education about doping and contact with dopers [44, 47].

### Performance enhancement attitude scale (PEAS)

Attitudes towards doping were operationalized as an individual’s predisposition towards the use of banned substances and methods set forth in the Code of the WADA [48]. Petróczi [45] proposed the PEAS, which quantitatively measures athletes’ attitudes towards doping, and a Korean translation of which by Kim and Kim [47] was used when collecting data. The original PEAS consisted of 17 items on a six-point Likert-type scale ranging from strongly disagree (1) disagree (2), slightly disagree (3), slightly agree (4), and agree (5) to strongly agree (6). No neutral response option was offered, and all items were scored in the same direction [49]. However, a Cronbach’s alpha value of 17-item version in this study was 0.66, and AR Nicholls, DJ Madigan and AR Levy [50] suggest that the 8-items version of the PEAS shows the best fit for adults. Therefore, only eight items were used with the other nine excluded. The total score for the eight items was used; a high score was defined as a permissive attitude towards doping, while a low score denotes a strict attitude [45]. This study showed a Cronbach’s alpha value for the 8-item version of the PEAS of 0.69.

### Perfectionism in sports scale (PSS)

To measure each athlete’s level of perfectionism in the sport, the PSS, proposed by Anshel and Eom [33] and translated into Korean by Kim et al. [51] was used. The PSS comprises 32 items on a five-point Likert-type scale, ranging from strongly disagree (1) to strongly agree (5) and divided into four sub-scales each including eight statements: parental criticism, coach’s criticism, concern over mistakes, and personal standards. Perfectionism was considered to be a personality characteristic that develops in part from parents’ relational influence [52], but Moran et al. [44] mention that for adult athletes, the extent of parental influence on sporting activities is minimal and the Cronbach’s alpha value of parental criticism under PSS was 0.48 in this study. Therefore, parental criticism was excluded from the data analysis based on the study of Tavakol and Dennick [53]. This study showed the Cronbach’s alpha value of the coach’s criticism, concern over mistakes, and personal standards of PSS were 0.78, 0.74, and 0.75, respectively.

### Perceived motivational climate in sport Questionnaire-2 (PMCSQ-2)

To assess athletes’ perceptions of the prominent motivational climate goal structures created by their coaches [54], the PMCSQ-2, proposed by Newton et al. [55] and translated into Korean by Kim et al. [51] was used. The Korean version of PMCSQ-2 was reported as being in a valid and reliable tool for measurement of motivational climate in Korean [56], and the study of Kim et al. [51] showed that Cronbach’s alpha of the higher-order scales under PMCSQ-2 ranged from 0.72 to 0.88. The PMCSQ-2 consists of 33 items on a five-point Likert-type scale, from strongly disagree (1) to strongly agree (5); it is divided into two higher-order scales, respectively covering ego-involving climate and task-involving climate [44, 55]. The former is represented by 16 items with three sub-scales: intra-team rivalry (3 items), unequal recognition (7 items), and punishment for mistakes (6 items). The latter consists of 17 items with three sub-scales of cooperative learning (4 items), effort/improvement (8 items), and important role (5 items). Cronbach’s alphas for ego- and task-involving climate of PMCSQ-2 were 0.91 and 0.88, respectively.

### Statistical analyses

Statistical analysis was performed using SPSS version 21.0 for Window (SPSS Inc., Chicago, IL, USA). Firstly, the Kolmogorov-Smirnov test was employed to test for normality of distribution of variables, and the null hypothesis for normality was accepted (*p* > 0.05). Hence, all data was expressed as means and standard deviations or frequencies and percentages, depending on the characteristic in question. Pearson’s correlation coefficients were used to identify correlations among PEAS, PSS, and PMCSQ-2 scores, whose reliability estimates were calculated using Cronbach’s alpha. Stepwise multiple linear regression was performed to investigate possible factors—perfectionism, motivational climate, and characteristics of athletes—significantly associated with athletes’ attitudes towards doping and to establish the best predictive regression model by excluding the variables without statistical significance. Categorical variables in the third group were converted into dummy variables. Variance inflation factor (VIF) was reported for the regression model, to identify the potential for collinearity among the independent variables [57]. Statistical significance was identified at *p* < 0.05.

## Results

Table 2 shows correlations among the PEAS, the sub-scales of PSS, and the higher-order scales of PMCSQ-2. Only the coach’s criticism of PSS showed a slight or weak correlation with concern over mistakes under PSS and ego-involving climate under PMCSQ-2 (Pearson’s correlation coefficients = 0.53 and 0.64, respectively) based on the study of Talyor [58].

**Table 2:** Pearson’s correlation coefficients among values

	PEAS	Coach’s criticism	Concern over mistakes	Personal standards	Task-involving climate	Ego-involving climate
PEAS	–					
Coach’s criticism	0.05	–				
Concern over mistakes	0.18*	0.53**	–			
Personal standards	−0.01	0.25**	0.45**	–		
Task-involving climate	−0.04	0.02	0.11	0.42**	–	
Ego-involving climate	0.16*	0.64**	0.45**	0.21**	−0.01	–

Note: Significant correlation: *p < 0.05, **p < 0.01

*PEAS* Performance Enhancement Attitude Scale

Table 3 shows the results of stepwise multiple linear regression investigating possible factors significantly associated with athletes’ attitudes towards doping which were obtained using 11 independent variables: age, gender, type of event, contact with doping users, knowledge on doping, education on doping, coach’s criticism, concern over mistakes, personal standards, task-involving climate, and ego-involving climate. This model was significant (*F*_(1, 196)_ = 6.364, *p* = 0.012), but it accounted for 3% of the variance in elite athletes’ PEAS. And it showed that the concern over mistakes of PSS was significantly related to PEAS (*β* = 0.177, *p* = 0.012).

**Table 3:** Stepwise multiple linear regression for attitude towards doping

	*B*	*SE*	*β*	*t*(*p*)	*R* ^*2*^	Adjusted *R* ^*2*^	*F*(*p*)	*df*	VIF
Dependent variable: PEAS					0.031	0.027	6.364 (0.012)	1, 196	
Independent variables:									
Constant	8.762	1.982		4.421 (0.001)					
Concern over mistakes	1.676	0.664	0.177	2.523 (0.012)					1.000

*PEAS* Performance Enhancement Attitude Scale, *VIF* variance inflation factor

## Discussion

This study aimed to investigate possible factors associated with attitudes towards doping among Korean national athletes who participated in the Rio 2016 Olympic Games. The results showed that the coach’s criticism of perfectionism has a slight or weak correlation with concern over mistakes under perfectionism and ego-involving climate under motivational climate. And the concern over the mistakes sub-scale of perfectionism was related to attitudes towards doping among Korean elite athletes, but weakly.

Perfectionism has been considered to be an adaptive trait to help achieve elite performance [26], but also as a maladaptive one to disturb it [25]. In fact, competitive athletes with higher perfectionism have been shown to be disproportionately prone to several dangerous and unhealthy behaviors, such as eating disorders and emotional problems [27, 28]; the review study of Flett and Hewitt [31] similarly showed that perfectionists tended to be under pressure to dope in order to gain a competitive advantage. The present study also indicated that perfectionism, especially concern over mistakes, was positively related to attitudes towards doping among Korean athletes. Bahrami et al. [30] conducted a study on the relationship between attitudes towards doping and perfectionism for bodybuilders, and mentioned that perfectionism, especially perfectionistic strivings and perfectionistic concerns, had positive correlations with positive attitudes towards doping. Zucchetti et al. [7] similarly conducted a study on the correlations of perfectionism and doping attitudes among Italian athletes, and showed that perfectionism was positively related to attitude towards doping. In addition, Madigan et al. [32] studied the relationship between perfectionism in sport and positive attitudes towards doping in junior athletes, and showed that the four aspects of perfectionism each separately influenced those attitudes. In other words, attitudes towards doping were positively correlated with parental pressure to be perfect but had a negative relationship with perfectionistic striving. According to these previous studies and the present study, which aspects/subscales of perfectionism play a role in attitudes toward doping seems to depend on age and type of sport played. Moreover, perfectionism in sports has been shown to be related to maximizing athletic potential or chance to win, in different proportions [35]. Thus, to verify the relationship between perfectionism and doping attitudes, a more coherent and organized study design considering athletes’ age, sport, and goal(s) is needed, and further study is required to explain the possible mediating effect of perfectionism on other independent variables related to attitudes towards doping.

Motivational climate was defined above as the psychological atmosphere in which physical education and/or sports activity takes place [59] and is important in explaining the quality of motivation exhibited by elite athletes [60]. As mentioned, the organization of the coaching environment (that is, motivational climate) has been shown to carry consequences for athletes’ ego or task goal orientation [61, 62], and this orientation is in turn significantly correlated with cheating and with attitudes towards doping [38]. Kavussanu [37] reported that athletes experience a mastery climate led to a task goal orientation rather than a performance climate led to an ego orientation and that a mastery climate is a negative predictor of attitudes towards doping. Allen et al. [16] showed that a coach-created mastery climate was negatively related to attitudes towards doping and mentioned that this protective influence of a mastery climate may become even more important. This study also showed that the ego-involving climate was (non-significantly) positively related to attitudes towards doping among Korean athletes, a result that suggests that how athletes define achievement in competitive situations is related to their doping attitudes. Therefore, athletics-related staff, especially coaches, who are an important social influence on athletes’ doping attitudes, intentions, and behavior [16], need to be educated on the importance of creating an appropriate motivational climate to prevent doping [44].

Not only were individual factors, such as perfectionism, related to doping attitudes and behaviors; social contextual factors, such as contact with peers who dope, were also [16]. Zucchetti et al. [7] similarly reported that athletes who have contact with dopers have more positive attitudes towards doping, and Petróczi and Aidman [17] and Wiefferink et al. [42] mentioned that a social network characterized by use of banned drugs may influence athletes’ attitudes towards doping. A previous study showed that Korean adult athletes who personally knew dopers had a more generous attitude toward doping compared to those who did not [47]. However, the present study showed that contact with dopers was only non-significantly related to attitudes towards doping among Korean athletes. This result was partly because few athletes (*n* = 10, 5.1%) were in contact with dopers because Korea is a relatively drug-free country.

WADA and national anti-doping agencies disseminate information and education on the dangers and consequences of doping in order to prevent doping, and these activities may indeed lead to increased knowledge and change doping behaviors [63]. However, Blank et al. [3] stated that highlighting the negative effects of doping has also been unsuccessful, that is, education concerned with information delivery does not appear to be successful. Previous studies also show that simple knowledge-transfer is not generally enough to change athletes’ beliefs and attitudes [13–15], and a recent meta-analysis similarly showed that these interventions were only partly effective [20]. The present study showed that many athletes have received education on doping and possess knowledge on it, but that these results do not statistically influence athletes’ doping attitudes. Therefore, to prevent doping, athletes at risk of doping should be identified [24], and integrative approaches that describe the dynamic interactions between risk factors and protective factors for doping should be employed [18].

According to Petróczi [64], while older athletes tend to achieve goals that are more performance oriented, making them more vulnerable to doping, younger athletes want to achieve goals that are more mastery-oriented making them less vulnerable. Also, male athletes were more susceptible to doping than female athletes [65]. Allen et al. [16] reported that athletes who compete in individual sports are higher ego orientations compared with team sports athletes, and may be more vulnerable to doping since they are more likely to be aiming to outperform other athletes. Kim and Kim [47] reported that Korean adult athletes’ attitudes towards doping are more permissive compared to those of adolescents, with no significant differences found between genders or even among sporting events. The results of the present study showed that Korean athletes’ attitudes towards doping did not depend on their age or gender or on the type of sporting event (individual or team) that they competed in; these results may be related to the relatively drug-free nature of Korean society. Similarly, the studies of Kim and Kim [47] and Moran et al. [44] showed that doping attitudes vary across four types of sports event; thus, further subdivision of sports events into types could provide more useful information to inform anti-doping strategies.

In this study, correlations were identified among various factors: perfectionism, motivational climate, education or knowledge on doping, and attitudes towards doping. However, these correlations were very low, and the cross-sectional design does not allow strong conclusions to be drawn on the causal relationships. To address this gap, future research should include other factors that may influence athletes’ doping attitudes, and a longitudinal and prospective cohort study would help determine the related causal relationships. Recently, various studies which aimed to identify perfectionism’s effects have claimed that the approaches to perfectionism are not one-dimensional but multi-dimensional such as adaptive/maladaptive, positive/negative and healthy/unhealthy [66]. In consideration of multidimensional operationalization of perfectionism, further studies will be conducted for identifying the relationship between perfectionism and attitudes towards doping. Additionally, this study involved only adult athletes who participated in the 2016 Olympic Games; attention should also be devoted to adolescent athletes are at risk of doping [67] to inform an anti-doping policy for adolescents.

## Conclusion

This study demonstrated that attitudes towards doping among Korean elite athletes who participated in the Rio 2016 Olympic Games were related to their perfectionism, especially their concern over mistakes, but the correlations involved were mostly very slight. Therefore, an effective anti-doping policy should meet athletes’ perfectionism, and more studies that identify other factors that influence athletes’ doping attitudes are needed.

## Abbreviations

KADA: Korea anti-doping agency
PEAS: Performance enhancement attitudes scale
PMCSQ-2: Perceived Motivational Climate in Sport Questionnaire-2
PSS: Perfectionism in Sports Scale
WADA: World anti-doping agency
